# The effect of different types of migration on symptoms of anxiety or depression and experience of violence among people who use or inject drugs in Kachin State, Myanmar

**DOI:** 10.1186/s12954-023-00766-1

**Published:** 2023-04-03

**Authors:** Khine Wut Yee Kyaw, Lucy Platt, Murdo Bijl, Sujit D. Rathod, Aung Yu Naing, Bayard Roberts

**Affiliations:** 1 Asian Harm Reduction Network (AHRN), Yangon, Myanmar; 2 Faculty of Public Health and Policy, London School of Hygiene and Tropical Medicine, London, UK; 3 Faculty of Epidemiology and Population Health, London School of Hygiene and Tropical Medicine, London, UK

**Keywords:** Forced displacement, Mental health conditions, Conflict-affected, Physical violence, Emotional violence

## Abstract

**Background:**

Evidence on the social determinants of mental health conditions and violence among people who inject or use drugs (PWUD) is limited, particularly in conflict-affected countries. We estimated the prevalence of symptoms of anxiety or depression and experience of emotional or physical violence among PWUD in Kachin State in Myanmar and examined their association with structural determinants, focusing on types of past migration (migration for any reason, economic or forced displacement).

**Materials:**

A cross-sectional survey was conducted among PWUD attending a harm reduction centre between July and November 2021 in Kachin State, Myanmar. We used logistic regression models to measure associations between past migration, economic migration and forced displacement on two outcomes (1) symptoms of anxiety or depression (Patient Health Questionnaire-4) and (2) physical or emotional violence (last 12 months), adjusted for key confounders.

**Results:**

A total of 406, predominantly male (96.8%), PWUD were recruited. The median age (IQR) was 30 (25, 37) years, most injected drugs (81.5%) and more commonly opioid substances such as heroin or opium (85%). Symptoms of anxiety or depression (PHQ4 ≥ 6) were high (32.8%) as was physical or emotional violence in the last 12 months (61.8%). Almost one-third (28.3%) had not lived in Waingmaw for their whole life (migration for any reason), 77.9% had left home for work at some point (economic migration) and 19.5% had been forced to leave home due to war or armed conflict (forced displacement). A third were in unstable housing in the last 3 months (30.1%) and reported going hungry in the last 12 months (27.7%). Only forced displacement was associated with symptoms of anxiety or depression [adjusted odds ratio, aOR 2.33 (95% confidence interval, CI 1.32–4.11)] and recent experience of violence [aOR 2.18 (95% CI 1.15–4.15)].

**Conclusion:**

Findings highlight the importance of mental health services integrated into existing harm reduction services to address high levels of anxiety or depression among PWUD, particularly among those who have been displaced through armed conflict or war. Findings reinforce the need to address broader social determinants, in the form of food poverty, unstable housing and stigma, in order to reduce mental health and violence.

**Supplementary Information:**

The online version contains supplementary material available at 10.1186/s12954-023-00766-1.

## Background

In 2020, an estimated 275 million people globally used drugs for non-medical purposes [1]. Greater use is reported in countries with higher levels of drug production, such as Afghanistan, Mexico and Myanmar, which share 95% of the global opium production [1]. Myanmar is also one of the world's leading amphetamine-type stimulants (ATS) producers. Methamphetamine is the most popular type of ATS in the market, and the use is in increasing trend [2].

Within Myanmar, drug production areas such as Kachin State, Northern Shan State and the Sagaing Region have the highest prevalence of drug use among the population [2, 3]. Elevated drug use in these contexts is thought to be attributable to increased availability of drugs and consequent changes in social norms. Increased drug use is thought to be a coping mechanism in response to increasing psychological distress and poor economic prospects as a result of historically decades of prolonged conflict between ethnic armed groups and state actors and forced displacement [4–9]. Evidence from Myanmar also shows a link between working in mining industries and opium, heroin or stimulant use, with drugs used as a way of coping with difficult working conditions [10]. For example, estimates of injecting drug use within Kachin State, where jade and gold mines are a common industry, are 5% among 15–49-year-old men compared to the national prevalence of 0.3% (15–64 years) [11–13].

There are also 912,000 internally displaced persons (IDPs) in Myanmar due to decades of conflict and violence, with the largest population in Kachin, Chin, Shan and Rakhine States [14]. This forced displacement can detrimentally affect health, creating disparities in social, economic and cultural opportunities, resulting in discrimination and racism, as well as reduced access to quality health care [15]. These factors are all linked with an increased risk of developing poor mental health and poorer outcomes [9, 16–18]. A meta-analysis reported that the prevalence of depression among displaced people, such as refugees, migrants, asylum seekers and IDPs, was 26.4%, and one-fourth of migrants suffer from depression globally [19].

Poor mental health conditions are a major source of mortality and morbidity globally [20]. People who use drugs can be more vulnerable to poor mental health due to homelessness, ill health and unemployment, compounded by stigma and the criminalised nature of drug use in most contexts [21, 22]. A global systematic review reported a high prevalence of severe depressive symptoms (42.0%), depression diagnosis (28.7%) and suicidal attempts (22.1%) among people who inject drugs (PWID) [23]. Evidence suggests that poor mental health can exacerbate drug-related outcomes, including increased risk of overdose, injecting-related injuries, injecting risk behaviours and acquiring HIV in people who use or inject drugs (PWUD) [23–25].

Studies have also documented heightened exposure to physical violence among people who use drugs across various settings [26, 27]. Aside from physical injury, violence can lead to poorer mental health, including post-traumatic stress disorder and anxiety, avoidance of health services and engagement in behaviours that can increase the risk of HIV acquisition [27–29]. Despite the documented need and the clear interplay between mental health, violence and HIV risk, screening and treatment of psychological conditions or violence have not been prioritised in the comprehensive harm reduction package recommended by the World Health Organization (WHO) and the implementation of mental health services in harm reduction interventions is limited [30]. Consequently, the experience of violence is neither routinely assessed nor documented, leading to missed opportunities for treatment and support.

The risk environment concept developed to understand drug-related harms examines how different types (physical, social, economic and political) and levels (macro and micro) of environmental influence shape health among people who use drugs, in line with broader efforts to address structural determinants of health [31]. Epidemiological evidence shows structural factors (e.g. law, housing, economic insecurity, stigma, displacement), community factors (e.g. policing practices, access to services) and individual behaviours (e.g. sharing needles/syringes) increase vulnerability to HIV and hepatitis C infection among PWID [31–34]. There is a growing body of evidence documenting the epidemiology of HIV among PWID in Myanmar, showing a high prevalence (35%) nationally and higher in Kachin State (54%) [12]. However, there has been little consideration of their structural determinants and a lack of evidence on the experience of violence or mental health conditions [35].

There is an urgent need to better understand the broader health and welfare of PWUD in Myanmar and their determinants to inform integrated prevention and intervention strategies. Drawing on data from a cross-sectional survey of PWUD in Kachin State, Myanmar, we estimate the prevalence of symptoms of depression or anxiety and experience of emotional or physical violence in the last 12 months. We examine their association with structural determinants, focusing on types of past migration (migration for any reason, economic or forced displacement).

## Methods

### Study design and setting

Kachin State has one of the largest populations of IDPs in Myanmar as well as migrants moving to work in gold and jade mines [36, 37]. United Nations agencies, international and local non-governmental organisations (NGOs) in collaboration with ethnic health organisations and faith-based organisations deliver services to IDPs, including the provision of shelter, non-food items, protection, health, education, nutrition, livelihoods, and access to clean water, sanitation and good hygiene practices. In contrast, services for migrant workers are limited. Harm reduction services are also provided by international and local NGOs in Kachin, and project sites are systematically divided among implementers to avoid services overlapping.

Since 2003, the Asian Harm Reduction Network (AHRN) has been implementing a comprehensive “one-stop-shop” harm reduction cascade of services in Kachin State and progressively expanded to Shan State and Sagaing Region. There are now 35 service delivery sites in three states and regions in Myanmar [38]. The Waingmaw centre was selected for this cross-sectional study because of high levels of drug use, including both injecting heroin and increased use of amphetamine and associated high prevalence of HIV (54%), HCV (85%) and other health needs among PWUD. There is also a sizeable mobile population and historically frequent outbreaks of conflict likely to produce particular health needs among the population, but which have not yet been researched [12]. Harm reduction services at Waingmaw service centre include psychosocial counselling, social support, health education, distribution of needles and syringes, condoms, facilitating access to methadone maintenance therapy (MMT), provision of HIV, TB and viral hepatitis prevention, diagnostics and treatment, overdose management, and other medical services through drop-in centres, key population service centres, mobile medical teams, outreach and community-based activities [39].

In Myanmar key population service centres are targeted towards men who have sex with men, PWID and sex workers and other vulnerable population including people who (non-injecting) use drugs and sexual partner of PWUD. They contain dedicated spaces for recreational activities and provide medical care such as primary health care, standard infectious and non-infectious disease screening, diagnosis and treatment. Screening and treatment services for most common psychological disorders such as depression and psychosis were first introduced to the key population service centre model in 2016 using the Mental Health Gap Action Programme (mhGAP), and it was expanded to many harm reduction facilities after 2017 [40]. Yet, mental health treatment options are limited, and some psychological disorders require referral to specialist care facilities. In Myanmar, public hospitals are the main sources of mental health services and drug treatment provision [2].

### Study population and sampling design

We conducted a clinic-based cross-sectional study using structured questionnaires in Waingmaw, Kachin State, Myanmar. The study was a collaboration between the AHRN and the London School of Hygiene and Tropical Medicine. PWUD, routinely identified by the clinic centre staff, who visited the clinic during the survey period between 16th July and 2nd November 2021, were invited to participate in the survey by AHRN's centre staff or a research interviewer. All available clients were invited to participate, and informed consent was obtained if they were eligible, interested, and had time to complete the questionnaire. Participants were eligible if they had: (i) used drugs (heroin or amphetamines) at least once in the last 3 months; (ii) were 14 years or older, and (iii) able to give consent. After completing the questionnaire, participants received 5000 MMK (~ 2.7 USD) in recognition for their time.

### Questionnaire and data collection

Following informed consent, interviews were conducted by four interviewers trained in research ethics, data collection procedures, and COVID-19 infection control measures in a private space at the centre. Interviewers administered a structured questionnaire in Burmese using a tablet (Open Data Kit V.1 28.4). Data were collected on demographics, health and service use, mental and physical health, drug use characteristics, violence and sexual practices, and stigma. Indicators were drawn from validated measures and other surveys among PWUD [41, 42]. The questionnaires were translated into Burmese by KWKY. We extracted HIV and HCV status from patient records in the clinic where available. If no test had been conducted or they had not been tested in the past 6 months, and their last test was negative, participants were offered to test and referred to the AHRN's on-site counsellor and laboratory technician.

### Outcomes

Our primary outcome was symptoms of anxiety or depression over the last 2 weeks measured through a validated composite measure (Patient Health Questionnaire-4, PHQ4) comprising two items from the Public Health Questionnaire focusing on depressed mood and loss of interest and two items from Generalised Anxiety Disorder scale (feeling anxious or inability to control worrying) [43]. The reliability of the PHQ4 with the study population was assessed using Cronbach's alpha, and it was 0.79, indicating an acceptable/strong level of internal reliability. We took the sum of the four items and made a binary outcome variable (Score 0–5 and ≥ 6) which has been validated as a reliable brief measure of depression or anxiety in other contexts [43].

As a secondary outcome, we measured experience of recent (last 12 months) either physical or emotional violence. The violence questions were developed based on the operational definition and questionnaire used in WHO multi-country study on women's health and domestic violence against women [42]. Physical violence was defined as being pushed, shoved, slapped, kicked, punched, choked, dragged, burnt, had a weapon against him/her, thrown something at him/her, or beaten him/her up. Emotional violence was defined as being called in a derogatory term (for example, opium-eater). A participant was classified as being subject to recent violence if they affirmed any of the violence questions.

### Co-variables

Our three key explanatory variables were defined as: (i) being a migrant (not living in Waingmaw township for the whole life); (ii) economic migration (ever left the home community for work); and (iii) forced displacement as an IDP (ever left the home community because of war or armed conflict). We considered stigma related to drug use and other structural factors for their hypothesised relationships with mental health and violence. 'Enacted Stigma' related to drug use was measured using the substance-use stigma mechanisms scale (SU-SMS) and concerned the experience of stigma within families and among health care workers [44]. The responses were given on a Likert scale of 1–5, with higher scores indicating greater endorsement of substance-use stigma. Average scores for sub-scales of enacted stigma for family members and health care workers were calculated, and stigma level was categorised using median scores.

Other factors considered included ethnicity (Kachin, Shan, Pa'O, Bamar, other), education (no school, primary, middle, high school, college), main source of income in the last 3 months (casual labourer, farming, office job, government job, army, shop or market worker, from parents/other relatives, from spouse, lover or friend's income, sex for money, no money to live on), housing status in the last 3 months (stable housing including own place, parents' house, rented private room and unstable housing including someone else's house, an IDP camp, sleeping on somebody's sofa/floor, squatting, having no fixed address, drug treatment institution, drug rehabilitation centre, jail or prison, and work-provided accommodation), and food insufficiency in the last 12 months (defined as being hungry and did not eat because you could not afford to buy enough food).

Patterns of drug use variables included type of drug use in the last 3 months (amphetamine-type stimulants (ATS) with or without opioid substance, opioid substances without ATS), mode (injecting, non-injecting) and location of drug use in the last 3 months (private place such as home and public places including places where they bought drugs, on the street, around the bushes, shooting galleries, public toilets, and workplace). Health care accessibility variables included type of current treatment (religious-based psyche-social support, government rehabilitation programme with overnight stay/ as day visit, private doctor, self-help/community support, methadone maintenance, counselling, NGO clinic), having outreach workers' visits in the last 12 months (yes/no).

### Statistical analyses

We examined univariable associations using logistic regression in separate models for each explanatory variable and each outcome. Variables significantly associated with outcomes (*P* < 0.05) in univariable analysis, and a priori confounders were included in multivariable models. For multivariable models, we adjusted for the following confounders for their association with migration and mental health and violence: age, housing, main income from farming in the last 3 months and type of drug used. We considered the presence of anxiety or depression symptoms and location of drug use variables as additional a priori confounders for the outcome of recent exposure to physical or emotional violence. We present crude odds ratio (OR) and adjusted odds ratio (aOR) with the 95% confidence interval (CI) produced from the models. All the analyses were done in Stata version 17.0 (Stata Corp, US).

## Results

Of the 1,237 unique PWUD who attended the service in Waingmaw during the recruitment period, some 417/1237 (33.8%) were screened and eligible for the study, while the remainder (*n* = 820, 66.2%) were not screened as they had then left clinics before meeting the interviewers. The comparative table presenting the characteristics of PWUD by screening status has been reported as Additional file 1. Of those who were screened, 406/417 (97.1%) clients provided consent. Ten of eleven recruits who declined consent reported time limitation as a reason, and one client said they were afraid of identifying as a drug user.

Characteristics of study participants are presented in Table 1. The median age of participants was 30 (IQR 25, 37) years. The majority were male (96.8%), 69.7% were of Kachin ethnicity, and a quarter (25.6%) had no school or primary school level education. The median age of first drug use was 20 (IQR 18–25) years, 81.5% had ever injected drugs, and over half (56.2%) injected drugs daily in the last 4 weeks. Heroin or opium was the main drug used (85.0%), but 15.0% used ATS with or without opioid substances in the last 3 months). Casual labour was the main source of income for 48.0% of participants, and 7.4% of respondents had no money in the last 3 months. One-third (28.1%) had ever had suicidal thoughts, and 13.2% had ever attempted suicide. Overall, 64.4% had antibodies to HIV and 78.7% had antibodies to HCV.

**Table 1:** Characteristics of study participants in Waingmaw, Myanmar, 2021

Characteristics	*n*	(Col %) or (IQR)
Total	406	
Demographic characteristics		
Median age (IQR)	30	(25, 37)
Male	393	(96.8)
Kachin ethnicity $	282	(69.5)
Education		
Primary school or no school	104	(25.6)
Middle school	167	(41.1)
High school and college	135	(33.3)
Main income from farming in the last 3 months	196	(48.3)
Drug use characteristics		
Median age of first drug use (IQR)	20	(18, 25)
Injecting drug use	331	(81.5)
Type of drug use in the last 3 months		
ATS with or without Opioid substance	61	(15.0)
Opioid substances without ATS	345	(85.0)
Receptive needle and syringe sharing in the last 3 months (*n* = 321)	112	(34.9)
Sharing pipes in the last 3 months (*n* = 339)	65	(19.2)
Location of drug use (*n* = 405)		
Private €	139	(34.3)
Public €€	266	(65.7)
Frequency of injection in the last 4 weeks (*n* = 386)		
Daily	163	(42.2)
Less than daily	127	(32.9)
No injection	96	(24.9)
Overdose in the last 12 months	46	(11.3)
Sexual risk behaviours		
Ever sold sex (*n* = 402)	12	(3.0)
Currently have an intimate partner in sexual relationship (*n* = 400)	164	(41.0)
Condom use in the last sex # (*n* = 403)	109	(27.0)
Social determinants		
Median of average stigma score within families σ (IQR)	2	(1, 3)
Median of average stigma score within health care providers *σ* (IQR)	1	(1, 1)
Non-stable housing in the last 3 months & (*n* = 399)	120	(30.1)
Ever slept rough (*n* = 404)	231	(57.2)
Ever squatted (*n* = 401)	12	(3.0)
Ever stayed in emergency accommodation (*n* = 406)	132	(32.5)
Stopped by police/anti-drugs squad in the last 12 months (*n* = 406)	109	(26.8)
Detained in the last 12 months (*n* = 406)	30	(7.4)
Went hungry in the last 12 months because could not afford food (*n* = 405)	112	(27.7)
Type of migration		
Migrant ^ (*n* = 406)	115	(28.3)
Economic migration ¥ (*n* = 398)	310	(77.9)
Forced displacement £ (*n* = 406)	79	(19.5)
Health care accessibility and treatment		
Ever taken drug treatment (*n* = 405)	220	(54.3)
Type of current treatment (*n* = 403)		
No current treatment	325	(80.6)
Methadone	40	(9.9)
Non-methadone treatment*	38	(9.4)
Had ORW visits in the last 12 months (*n* = 392)	82	(20.9)
Health outcomes		
Recent experience of emotional violence µ (*n* = 403)	246	(61.0)
Recent experience of physical violence ¶ (*n* = 404)	37	(9.2)
Recent experience of physical or emotional violence (*n* = 406)	251	(61.8)
Ever had suicidal ideation δ (*n* = 405)	114	(28.1)
Ever attempted suicide ф (*n* = 401)	53	(13.2)
Presence of symptoms of depression or anxiety (*n* = 406): PHQ4 ≥ 6	133	(32.8)
HIV result (*n* = 396): positive	255	(64.4)
HCV result (*n* = 342): positive	269	(78.7)
HIV and HCV result (*n* = 342): positive	150	(55.6)

n = number, Col% = column percentage, IQR = interquartile range, ATS = amphetamine-type stimulants, ORW = outreach workers, PHQ4 = patient health questionnaire-4, HIV = human immunodeficiency virus, HCV = hepatitis C virus

$Non-Kachin ethnicity contained Shan, Bamar, Chinese, Gawrakha, Karen, Mon, Naga, and Rakhine ethnicities

€Location of drug was private when clients usually used drugs at home in the last 3 months

€€Location of drug use was public when study participants usually used drugs at the places where the drugs were bought, on the street, around the bushes, in the public toilets, at work, in the forest, on the riverbank, at the farm and the religious drug treatment centre in the last 3 months

#Condom use in the last sex refers to reported condom use in the latest vaginal or anal, or oral sex

σSigma score can be interpreted as 1 = Never, 2 = Not often, 3 = somewhat often, 4 = Often, 5 = Very Often

&Non-stable housing refers to living in work-provided accommodation or living in someone's house or an internally displaced persons camp, no fixed address, drug treatment institution, drug rehabilitation centre, or jail in the last 3 months

^Migrant was defined as those who had not been living in Waingmaw for their whole life

¥Economic migration refers to being had ever migrated for work

£Forced displacement refers to being had ever migrated because of war or armed conflict

*Non-methadone treatment refers to religious-based psychosocial support, counselling, and non-methadone treatment at NGO clinics

µRecent experience of emotional violence refers to being called in a derogatory term in the last 12 months

¶Recent experience of physical violence refers to being physically abused in the last 12 months

δSuicidal ideation refers to ever having thought about ending life or hurting oneself

фAttempted suicide refers to ever being tried to kill oneself

### Other social determinants

Approximately a third of participants (27.7%) had felt hungry in the last 12 months or lived in unstable housing (30.1%) in the last 3 months. Within the previous 12 months of the survey, 26.8% were stopped by police or anti-drugs squad, and 7.6% were detained. The most common reason for the arrest was drug possession or drug use (38.3%), followed by theft or robbery (36.2%). The median average stigma score experienced within families was 2 (IQR 1–3), while for experience from health care workers was 1 (IQR 1–1). More than half (54.3%) of respondents had ever taken any treatment to modify or reduce or stop drug use, and 35.5% were on treatment at the time of the survey, with 51.3% (40/78) on methadone. In the last 12 months prior to the survey, 20.9% had outreach workers' visits, 74.0% had ever had an HIV test, and 57.3% had ever had an HCV test. HIV, HCV and dual infections positivity were 64.4%, 78.7% and 55.6%.

### Migration

Overall, 28.3% of participants were identified as migrants (defined as those who had not been living in Waingmaw for their whole life), 77.9% reported economic migration (defined as those who had ever left the home community for work), and 19.5% had ever experienced forced displacement (defined as those who had ever left the home community because of war or armed conflict).

#### Violence and mental health

Overall, 61.0% of participants (*n* = 246/403) had experienced recent emotional violence. The most common perpetrators were family members (*n* = 131, 33.5%), strangers (*n* = 96, 24.6%) and friends (*n* = 91, 23.3%). Twenty-five participants (6.4%) reported intimate partner as perpetrator of emotion violence. Less than 10% of study participants experienced recent physical violence (*n* = 37/404, 9.2%). The most common perpetrators were strangers (*n* = 9, 23.1%), family members (*n* = 8, 20.5%), friends (*n* = 7, 18.0%) and police (*n* = 6, 15.4%). There was no participant who reported intimate partner as the perpetrator of physical violence. Almost two-thirds (*n* = 251, 61.8%) of respondents reported recent experience of physical or emotional violence. More than two-thirds (67.2%) of respondents had symptoms of anxiety or depression (PHQ4 score 6 or more).

### Effect of migration on symptoms of anxiety or depression

We found no evidence that past migration was associated with symptoms of anxiety or depression [aOR 2.1 (95% CI 0.86–5.11)]. Among other social determinants, there was evidence that people who had experiencing stigma within families (≥ 2 average stigma score) had 1.7 times higher odds of symptoms of anxiety or depression [aOR 1.67 (95% CI 1.02–2.72)], relative to those who had experiences less stigma.

We found no evidence that economic migration was associated with symptoms of anxiety or depression [aOR 0.95 (95% CI 0.53–1.71)]. There was some evidence that experiencing emotional violence in the last 12 months was associated with increased odds of symptoms of anxiety or depression [aOR 1.70 (95% CI 1.01–2.84)].

We found evidence that people who had experienced forced displacement had 2.3 times higher odds of symptoms of anxiety or depression [aOR 2.33 (95% CI 1.32–4.11)], relative to those who had not experienced forced displacement. Participants experiencing higher stigma within families had higher odds of having symptoms of anxiety or depression [aOR 1.73 (95% CI 1.05–2.83)].

In all three models, there was evidence that going hungry in the last 12 months was associated with increased odds of symptoms of anxiety or depression, whereas participants reporting farming as a main source of income in the last 3 months had lower odds of symptoms of anxiety or depression than those whose income was from other sources (mining, logging, driving vehicles, skilled works and selling) (Table 2). The separate analysis of depression and anxiety is presented in Additional file 1: Tables S1, S2 and S3.

**Table 2:** Associations of characteristics with the presence of symptoms of anxiety or depression among study participants

Characteristics	Total	Col %	Anxiety or depression δδ		Unadjusted		Migrant^		Economic migration ¥			Forced displacement £		
			n	Row %	OR	(95% CI)	aOR	(95% CI)	aOR	(95% CI)		aOR		(95% CI)
Total	406		133	(32.8)										
Age group														
≤ 24	90	(22.2)	33	(36.7)	ref		ref		ref			ref		
25–34	182	(44.8)	55	(30.2)	0.75	(0.44–1.27)	0.8	(0.44–1.45)	0.82	(0.45–1.49)		0.79		(0.43–1.44)
35–44	106	(26.1)	38	(35.8)	0.97	(0.54–1.73)	1.13	(0.59–2.20)	1.19	(0.62–2.32)		1.12		(0.57–2.18)
≥ 45	28	(6.9)	7	(25.0)	0.58	(0.22–1.50)	0.84	(0.28–2.47)	0.86	(0.29–2.52)		0.91		(0.31–2.68)
Ethnicity														
Non-Kachin$	123	(30.3)	39	(31.7)	ref									
Kachin	283	(69.7)	94	(33.2)	1.07	(0.68–1.69)								
Education														
Primary school or no school	104	(25.6)	27	(26.0)	ref		ref		ref			ref		
Middle school	167	(41.1)	54	(32.3)	1.36	(0.79–2.35)	1.18	(0.64–2.18)	1.14	(0.61–2.11)		1.32		(0.70–2.48)
High school and college	135	(33.3)	52	(38.5)	**1.79**	**(1.02–3.12)**	1.41	(0.75–2.66)	1.34	(0.71–2.53)		1.59		(0.83–3.02)
Main income from farming in the last 3 months														
Yes	196	(48.3)	54	(27.6)	**0.63**	**(0.41–0.96)**	**0.56**	**(0.34–0.9)**	**0.53**	**(0.33–0.86)**		**0.52**		**(0.32–0.84)**
No	210	(51.7)	79	(37.6)	ref		ref		ref			ref		
Type of drug use in the last 3 months														
ATS with or without Opioid substance	61	(15.0)	15	(24.6)	ref		ref		ref			ref		
Opioid substances without ATS	345	(85.0)	118	(34.2)	1.59	(0.85–2.97)	1.76	(0.89–3.51)	1.77	(0.88–3.59)		1.68		(0.84–3.36)
Ever injected drugs														
Yes	331	(81.5)	107	(32.3)	0.9	(0.53–1.53)								
No	75	(18.5)	26	(34.7)	ref									
Detained in the last 12 months (*n* = 406)														
Yes	30	(7.4)	11	(36.7)	1.21	(0.56–2.61)								
No	376	(92.6)	122	(32.4)	ref									
Stopped by police/anti-drugs squad in the last 12 months (*n* = 406)														
Yes	109	(26.8)	37	(33.9)	1.08	(0.68–1.71)								
No	297	(73.2)	96	(32.3)	ref									
Housing status in the last 3 months (*n* = 399)														
Stable housing	279	(69.9)	92	(33.0)	ref		ref		ref			ref		
Non-stable housing&	120	(30.1)	38	(31.7)	0.94	(0.60–1.49)	0.72	(0.43–1.22)	0.79	(0.47–1.33)		0.72		(0.43–1.21)
Felt hungry the last 12 months (*n* = 405)														
Yes	112	(27.7)	59	(52.7)	**3.29**	**(2.09–5.19)**	**2.83**	**(1.70–4.69)**	**2.86**	**(1.70–4.80)**		**2.89**		**(1.73–4.81)**
No	293	(72.3)	74	(25.3)	ref		ref		ref			ref		
Recent experience of emotional violence *µ* (*n* = 403)														
Yes	246	(60.7)	95	(38.6)	**2.04**	**(1.30–3.20)**	1.63	(0.98–2.71)	**1.7**	**(1.01–2.84)**		1.46		(0.87–2.46)
No	157	(38.8)	37	(23.6)	ref		ref		ref			ref		
Recent experience of physical violence ¶ (*n* = 404)														
Yes	37	(9.2)	18	(48.6)	**2.08**	**(1.05–4.10)**	1.24	(0.56–2.75)	1.48	(0.66–3.32)		1.23		(0.55–2.73)
No	367	(90.8)	115	(31.3)	ref		ref		ref			ref		
HIV result (*n* = 396)														
Negative	141	(35.6)	50	(35.5)	ref									
Positive	255	(64.4)	77	(30.2)	0.79	(0.51–1.22)								
Had ORW visits in the last 12 months (*n* = 392)														
Yes	82	(20.9)	26	(31.7)	ref									
No	310	(79.1)	99	(31.9)	1.01	(0.60–1.70)								
Type of current treatment (*n* = 403)														
No current treatment	325	(80.6)	109	(33.5)	ref									
Methadone	40	(9.9)	10	(25.0)	0.66	(0.31–1.40)								
Non-methadone treatment*	38	(9.4)	14	(36.8)	1.16	(0.58–2.32)								
Average stigma score within families *σ* (*n* = 406)														
Between 1 and 2	213	(52.5)	50	(23.5)	ref		ref		ref			ref		
Between 2 and 5	193	(47.5)	83	(43.0)	**2.46**	**(1.61–3.77)**	**1.67**	**(1.02–2.72)**	1.56	(0.95–2.56)		**1.73**		**(1.05–2.83)**
Average stigma score within health care providers *σ* (*n* = 406)														
Score 1	380	(93.6)	118	(31.1)	ref		ref		ref			ref		
More than 1	26	(6.4)	15	(57.7)	**3.03**	**(1.35–6.79)**	2.1	(0.86–5.11)	1.94	(0.78–4.83)		2.3		(0.93–5.64)
Being migrant^ (*n* = 406)														
Yes	115	(28.3)	44	(38.3)	1.41	(0.90–2.21)	1.32	(0.79–2.22)						
No	291	(71.7)	89	(30.6)	ref		ref							
Economic migration ¥ (*n* = 398)														
Yes	310	(77.9)	106	(34.2)	1.39	(0.82–2.34)			0.95	(0.53–1.71)				
No	88	(22.1)	24	(27.3)	ref				ref					
Forced displacement £ (*n* = 406)														
Yes	79	(19.5)	39	(49.4)	**2.42**	**(1.46–3.99)**						**2.33**	**(1.32–4.11)**	
No	327	(80.5)	94	(28.7)	ref							ref		

n = number, ref = reference category, col% = column percentage, Row% = row percentage, OR = unadjusted odds ratio, aOR = adjusted odds ratio, 95% CI = 95% confidence interval, ATS = amphetamine-type stimulants, HIV = human immunodeficiency virus, HCV = hepatitis C virus, statistically significant associations are indicated in bold

δδPresence of symptoms of anxiety or depression was defined as having the PHQ4 score of 6 or more than 6

$Non-Kachin ethnicity contained Shan, Bamar, Chinese, Gawrakha, Karen, Mon, Naga, and Rakhine ethnicities

&Non-stable housing refers to living in work-provided accommodation or living in someone's house or an internally displaced persons camp, no fixed address, drug treatment institution, drug rehabilitation centre or in jail in the last 3 months

*Non-methadone treatment refers to religious-based psychosocial support, counselling, and treatment at NGO clinics. Methadone was provided only in the public facilities at the survey site

σSigma score can be interpreted as 1 = Never, 2 = Not often, 3 = somewhat often, 4 = Often, 5 = Very Often

^Migrant was defined as those who had not been living in Waingmaw for their whole life, and the adjusted model used migrant as a key exposure variable

¥Economic migration refers to those who had ever migrated for work, and the adjusted model used economic migration as a key exposure variable

£Forced displacement refers to those who had ever migrated because of war or armed conflict, and the adjusted model used forced displacement as key exposure variable

µRecent experience of emotional violence refers to being called in a derogatory term in the last 12 months

¶Recent experience of physical violence refers to being physically abused in the last 12 months

### Effect of migration on physical or emotional violence

We found no evidence that past migration was associated with recent physical or emotional violence [aOR 0.89 (95% CI 0.53–1.48)]. However, there was evidence that the presence of symptoms of anxiety or depression [aOR 1.77 (95% CI 1.07–2.94)] was associated with increased odds of physical or emotional violence.

We found no evidence that economic migration was associated with recent physical or emotional violence [aOR 1.21 (95% CI 0.70–2.07)]. There was evidence that those who had symptoms of anxiety or depression had greater odds of recent physical or emotional violence [aOR 1.87 (95% 1.12–3.12)].

There was evidence that forced displacement was associated with higher odds of exposure to physical or emotional violence [aOR 2.18 (95% CI 1.15–4.15)]. In all three models, participants reporting high stigma scores within families had higher odds of physical or emotional violence, while being on methadone was associated with reduced odds of physical or emotional violence (Table 3).

**Table 3:** Associations of characteristics with recent physical or emotional violence reported by the study participants

Characteristics	Total	Col %	Recent physical or emotional violence $$		Unadjusted		Migrant^		Economic migration¥		Forced displacement£	
			*n*	Row %	OR	(95% CI)	aOR	(95% CI)	aOR	(95% CI)	aOR	(95% CI)
Total	406		251	(61.8)								
Age group												
≤ 24	90	(22.2)	62	(68.9)	ref		ref		ref		ref	
25–34	182	(44.8)	110	(60.4)	0.69	(0.40–1.18)	0.76	(0.42–1.39)	0.76	(0.42–1.40)	0.72	(0.40–1.32)
35–44	106	(26.1)	65	(61.3)	0.72	(0.40–1.30)	0.95	(0.49–1.86)	0.92	(0.47–1.80)	0.94	(0.48–1.85)
≥ 45	28	(6.9)	14	(50.0)	0.45	(0.19–1.07)	0.85	(0.32–2.29)	0.77	(0.28–2.09)	0.82	(0.31–2.20)
Ethnicity												
Non-Kachin$	123	(30.3)	66	(53.7)	ref		ref		ref		ref	
Kachin	283	(69.7)	185	(65.4)	**1.63**	**(1.06–2.51)**	1.44	(0.88–2.35)	1.42	(0.87–2.33)	1.35	(0.83–2.22)
Education												
Primary school or no school	104	(25.6)	58	(55.8)	ref							
Middle school	167	(41.1)	103	(61.7)	1.28	(0.78–2.10)						
High school and college	135	(33.3)	90	(66.7)	1.59	(0.94–2.69)						
Main income from farming in the last 3 months												
Yes	196	(48.3)	118	(60.2)	0.88	(0.59–1.31)	1.12	(0.70–1.78)	1.2	(0.75–1.92)	1.13	(0.71–1.81)
No	210	(51.7)	133	(63.3)	ref		ref		ref		ref	
Type of drug use in the last 3 months												
ATS with or without Opioid substance	61	(15.0)	37	(60.7)	ref		ref		ref		ref	
Opioid substances without ATS	345	(85.0)	214	(62.0)	1.06	(0.61–1.85)	0.94	(0.49–1.8)	0.95	(0.49–1.84)	0.9	(0.47–1.75)
Ever injected drugs												
Yes	331	(81.5)	216	(65.3)	**2.15**	**(1.29–3.56)**	1.71	(0.96–3.07)	1.78	(0.99–3.22)	**1.96**	**(1.07–3.58)**
No	75	(18.5)	35	(46.7)	ref		ref		ref		ref	
Detained in the last 12 months (*n* = 406)												
Yes	30	(7.4)	24	(80.0)	**2.63**	**(1.05–6.58)**						
No	376	(92.6)	227	(60.4)	ref							
Stopped by police or anti-drugs squad in the last 12 months (*n* = 406)												
Yes	109	(26.8)	75	(68.8)	**1.52**	**(0.95–2.42)**	1.31	(0.78–2.20)	1.25	(0.74–2.12)	1.26	(0.75–2.13)
No	297	(73.2)	176	(59.3)	ref		ref		ref		ref	
Housing status in the last 3 months (*n* = 399)												
Stable housing	279	(69.9)	170	(60.9)	ref		ref		ref		ref	
Non-stable housing&	120	(30.1)	76	(63.3)	1.11	(0.71–1.72)	1.23	(0.74–2.05)	1.13	(0.67–1.9)	1.13	(0.68–1.89)
Felt hungry the last 12 months (*n* = 405)												
Yes	112	(27.7)	77	(68.8)	1.53	(0.96–2.42)						
No	293	(72.3)	173	(59.0)	ref							
HIV result (*n* = 396)												
Negative	141	(35.6)	88	(62.4)	ref							
Positive	255	(64.4)	159	(62.4)	1.00	(0.65–1.53)						
Had ORW visits in the last 12 months (*n* = 392)												
Yes	82	(20.9)	56	(68.3)	0.7	(0.42–1.17)						
No	310	(79.1)	186	(60.0)	ref							
Type of current treatment (*n* = 403)												
No current treatment	325	(80.6)	206	(63.4)	ref		ref		ref		ref	
Methadone	40	(9.9)	16	(40.0)	**0.39**	**(0.20–0.75)**	**0.38**	**(0.18–0.79)**	**0.39**	**(0.18–0.82)**	**0.38**	**(0.18–0.80)**
Non-methadone treatment*	38	(9.4)	28	(73.7)	1.62	(0.76–3.45)	1.4	(0.62–3.17)	1.48	(0.65–3.36)	1.28	(0.56–2.92)
Location of drug use (*n* = 405)												
Private €	139	(34.3)	85	(61.2)	ref		ref		ref		ref	
Public €€	266	(65.7)	165	(62.0)	1.04	(0.68–1.58)	0.95	(0.59–1.53)	0.92	(0.57–1.49)	0.88	(0.54–1.43)
Presence of symptoms of depression or anxiety (*n* = 406)												
PHQ4 < 6	273	(67.2)	154	(56.4)	ref		ref		ref		ref	
PHQ4 ≥ 6	133	(32.8)	97	(72.9)	**2.08**	**(1.33–3.27)**	**1.77**	**(1.07–2.94)**	**1.87**	**(1.12–3.12)**	1.6	(0.96–2.67)
Average stigma score within family *σ* (*n* = 406)												
Between 1 and 2	213	(52.5)	103	(48.4)	ref		ref		ref		ref	
Between 2 and 5	193	(47.5)	148	(76.7)	**3.51**	**(2.29–5.39)**	**3.1**	**(1.95–4.92)**	**3.17**	**(1.98–5.07)**	**3.09**	**(1.94–4.94)**
Average stigma score within health care providers *σ* (*n* = 406)												
Score 1	380	(93.6)	232	(61.1)	ref							
More than 1	26	(6.4)	19	(73.1)	1.73	(0.71–4.22)						
Being migrant^ (*n* = 406)												
Yes	115	(28.3)	72	(62.6)	1.05	(0.67–1.64)	0.89	(0.53–1.48)				
No	291	(71.7)	179	(61.5)	ref		ref					
Economic migration ¥ (*n* = 398)												
Yes	310	(77.9)	196	(63.2)	1	(0.89–2.31)			1.21	(0.70–2.07)		
No	88	(22.1)	48	(54.5)	ref				1			
Forced displacement £ (*n* = 406)												
Yes	79	(19.5)	60	(75.9)	**2.25**	**(1.28–3.94)**					**2.18**	**(1.15–4.15)**
No	327	(80.5)	191	(58.4)	ref						ref	

n = number, ref = reference category, col% = column percentage, Row% = row percentage, OR = unadjusted odds ratio, aOR = adjusted odds ratio, CI = confidence interval, ATS = amphetamine-type stimulants, HIV = human immunodeficiency virus, statistically significant associations are indicated in bold

$$Recent physical or emotional violence refers to being physically abused or called in a derogatory term in the last 12 months

$Non-Kachin ethnicity contained Shan, Bamar, Chinese, Gawrakha, Karen, Mon, Naga and Rakhine ethnicities

&Non-stable housing refers to living in work-provided accommodation or living in someone's house or an internally displaced persons camp, no fixed address, drug treatment institution, drug rehabilitation centre or in jail in the last 3 months

*Non-methadone treatment refers to religious-based psychosocial support, counselling and treatment at NGO clinics. Methadone was provided only in the public facilities at the survey site

σSigma score can be interpreted as 1 = Never, 2 = Not often, 3 = somewhat often, 4 = Often, 5 = Very Often

^Migrant was defined as those who had not been living in Waingmaw for their whole life, and the adjusted model used migrant as a key exposure variable

¥Economic migration refers to those who had ever migrated for work, and the adjusted model used economic migration as a key exposure variable

£Forced displacement refers to those who had ever migrated because of war or armed conflict, and the adjusted model used forced displacement as a key exposure variable

€Drug use in private means study participants usually used drugs at home in the last 3 months of the survey date

€€Drug use in public means study participants usually used drugs at the places where the drugs were bought, on the street, around the bushes, in the public toilets, at work, in the forest, on the riverbank, in the farm and at the religious drug treatment centre in the last 3 months

## Discussion

To the best of our knowledge, this is the first study reporting mental health and recent physical or emotional violence among PWUD and their association with migration and other social determinants in Myanmar. We observed a high prevalence of symptoms of anxiety or depression (32.8%). One in ten PWUD had experienced physical violence in the last year, and six in ten had experienced emotional violence in the form of verbal abuse. In an examination of the effect of different forms of migration on mental health and violence, only forced displacement was associated with increased odds of both symptoms of depression or anxiety and experience of physical or emotional violence. We also found evidence that experiencing stigma from family members was associated with both symptoms of anxiety or depression and physical and emotional violence in all models.

One-third of participants reported symptoms of anxiety or depression in our study, almost a third had ever had suicidal thoughts, and 13% had ever attempted suicide. Prevalence of symptoms of anxiety or depression is in line with other evidence from a systematic review, reporting the presence of severe depression symptoms in 42.0% of PWID, and Ukraine's study on PWID living with HIV, showing poor mental health conditions among PWID and highlights the imperative to include mental health services within the harm reduction cascade of service delivery [23, 45]. Besides, the link between mental health conditions and substance use disorder was reported elsewhere [46, 47]. Findings also highlight the precarious conditions in which PWUD live in Kachin, a third of whom had the experience of unstable housing in the last 3 months and food insecurity in the last 12 months and a quarter of whom have been stopped by the police or anti-drugs squad in the last 12 months, indicators of extreme marginalisation linked to poor mental health in other settings [21, 48].

The link between poor mental health and conflict has been well established. A global review estimated the prevalence of depression to be 10.8% and any anxiety disorder at 21.7% among people affected by conflict; among our sample 1 in three participants reported symptoms of anxiety or depression, and prevalence and odds were higher among those who had been displaced due to armed conflict or war [16]. Due to the cross-sectional design of the study, it is impossible to establish the temporal sequencing between displacement, initiation into drug use and the onset of poor mental health. However, findings suggest that the already high levels of poor mental health conditions among people who use drugs are attenuated among those displaced as a result of armed conflict and war. Evidence from other regions shows that drug use among people living in or displaced from conflict-affected countries is more frequent due to increased drug availability, changing social norms or structure, lacking economic opportunities, and coping with trauma [4]. Mental health services should be made available widely in harm reduction settings, and health facilities providing care to PWUD in conflict-affected areas should be person-tailored to individual needs of internally displaced persons, including psychological support, income generation, facilitating social integration process and referral mechanisms for coping with ongoing stressors and improving access to essential health care [49].

We failed to find any evidence to support or refute our hypothesis that past experience of migration (for any reason) or migration for work was associated with poor mental health or experience of emotional or physical violence. This may be related to the imprecision of the question that failed to capture specific aspects of vulnerability faced by these types of migration and heterogeneous nature of characteristics among them [50]. Evidence shows a complex relationship between migration and health, with health outcomes confounded by pre-migration experiences, socio-economic status, the availability and agency of host communities for migrants that could possibly lead to healthier conditions and reduced isolation [51]. In contrast with those forcibly displaced who are more likely to live in temporary housing or emergency accommodation in often overcrowded conditions and less able to integrate with host communities or access to health services [15, 19]. Further research in Myanmar is needed to understand the additional vulnerabilities that forced displacement as opposed to economic migration, has for PWUD.

The findings add to growing evidence of the interplay between stigma, mental health, and violence [7, 52, 53]. More than half of the participants had experienced physical or emotional violence in the last 12 months, and this was associated with symptoms of anxiety or depression. Our finding of more stigma experienced within families than in healthcare settings is possibly a consequence of our sampling strategy that focused on a harm reduction service where staff are likely to be more sensitised to drug use. We found evidence that experiencing stigma from family members was associated with both symptoms of anxiety and depression and recent violence. Family members' behaviours toward PWUD, who were found to be the most common perpetrators of emotional violence in our study, could also be linked to social stigma and the considerable psychological distress they experience [54]. It also may reflect stigma toward drug use in society more broadly, where widespread poverty intersects with long-standing conflict, drug production and drug use, creating conditions where poor health thrives, as is most clearly evident in the large-scale epidemics of HIV linked to drug injecting [12].

The gross domestic product per capita income of a quarter of the Myanmar population was below the poverty line. Besides, food insecurity is high in conflict-affected border states, including Kachin state, due to increasing market prices of rice and cooking oil and compromised life and livelihoods of the population [55, 56]. Other evidence shows a clear relationship between drug use-related stigma and mental health conditions linked with the experience of violence, which can increase vulnerability to violence, particularly among women [53, 57], while stigma also negatively affects overall health and the uptake of health services [58, 59].

Addressing widespread stigma towards people who use drugs is complicated and requires substantial cultural changes within communities and society more broadly. Efforts should be undertaken include raising awareness about the availability and effectiveness of drug treatment services, the promotion of alternatives to imprisonment for drug offences in recognition of the negative social and health consequences for PWUD, their families and communities, as well as reducing the negative portrayal of drug use alongside violence and crime in social and mainstream media [54, 60]. Longer-term initiatives are also required, such as addressing poverty and homelessness that increase stigma towards PWUD as well as perpetuating drug use [61].

We found evidence of an association between methadone and reduced odds of physical and emotional violence. While we are unable to elucidate the pathways between methadone and emotional or physical violence, evidence from a linked qualitative study among PWUD in Bhamo and Waingmaw in Kachin State suggests that the provision of methadone plays an important role in relieving economic pressure, affording people opportunities to work rather than finding and paying for their own methadone or other drugs and exacerbating already precarious livelihoods [62].

The study had several limitations. The study was cross-sectional in nature and cannot determine the temporal relationship or causality. The use of convenience sampling of clinic attendance in place of the planned respondent-driven sampling was necessary due to security constraints but limits the inferences we can make on the representativeness of findings to other PWUD in Kachin state, particularly to the large population living in rural areas who will have reduced access to clinics in townships. Findings are also drawn from self-reports which may be subject to reporting bias, particularly given the sensitive nature of questions in relation to mental health and violence.

Our study focused on measuring three forms of migration experience as a primary exposure, and models adjust for confounders associated with these exposures and our outcomes. We report the effect estimates for confounders and other covariates associated with the outcomes but note the limitations of this approach and that other unmeasured factors may confound these estimates [63]. These are reported here given the exploratory nature of the analysis and in the absence of research to inform interventions among a highly marginalised group but should be interpreted with caution.

Reports of physical violence were lower than anticipated (9.2%), given the context of conflict. Further research is needed to corroborate and understand the experience of violence. Measures used for assessing mental health outcomes reported on symptoms, and they are not diagnostic. They have also not been psychometrically tested and validated in Myanmar; however, they have been used across different settings, and findings suggest high internal reliability in our study (Cronbach's *α* = 0.79) [64, 65]. We could not recruit many women into the study, reflecting the gender profile of PWUD attending the centre, who are predominantly male and the highly stigmatised and hidden nature of drug use among women [66]. There is an urgent need for more research to understand the health needs among women who use or inject drugs in the region to inform appropriate services, especially in relation to violence [67].

## Conclusions

The high prevalence of symptoms of anxiety or depression and physical or emotional violence in Kachin State supports the need to integrate harm reduction services with interventions to address mental health and violence. Findings point to the need for expanded mental health services in Myanmar among PWUD, particularly with internally displaced persons. There is a need for longer-term policy and social changes to address broader determinants of the marginalisation of PWUD, including addressing poverty, stigma and forced displacement. In the short term, our findings highlight opportunities to address the immediate health needs of PWUD in relation to mental health and violence within harm reduction services to improve the health and well-being of this highly marginalised population.

## Supplementary Information

**Additional file 1: Table S1** Characteristics of PWUD who were screened and not screened during their visit to the Waingmaw AHRN clinic during the survey period.** Table S2** Factors associated with symptoms of depression among survey clients (*n* = 406).** Table S3** Factors associated with symptoms of anxiety among survey clients (*n* = 406)

## Data Availability

The data sets used and/or analysed during the current study are available from the corresponding author upon reasonable request.

## Abbreviations

AHRN: Asian Harm Reduction Network
aOR: Adjusted odds ratio
ATS: Amphetamine-type stimulants
CI: Confidence interval
col: Column
HCV: Hepatitis C virus
HIV: Human Immunodeficiency virus
IDP: Internally displaced person
IQR: Interquartile range
mhGAP: Mental Health Gap Action Programme
MMT: Methadone maintenance therapy
n: Number
NGO: Non-governmental organisation
OR: Unadjusted odds ratio
ORW: Outreach worker
PHQ-4: Patient Health Questionnaire-4
PWID: People who inject drugs
PWUD: People who use or inject drugs
ref: Reference
UK: United Kingdom
USD: United States Dollar
WHO: World Health Organization
