# Supplementary material for: The effect of different types of migration on symptoms of anxiety or depression and experience of violence among people who use or inject drugs in Kachin State, Myanmar
Source: Harm Reduct J. 2023 Apr 3;20:45. doi: 10.1186/s12954-023-00766-1 (PMC10068727; doi:10.1186/s12954-023-00766-1)
Supplement: Supplementary file 1 — Additional file 1: Table S1 Characteristics of PWUD who were screened and not screened during their visit to the Waingmaw AHRN clinic during the survey period. Table S2 Factors associated with symptoms of depression among survey clients (n = 406). Table S3 Factors associated with symptoms of anxiety among survey clients (n = 406) [file 12954_2023_766_MOESM1_ESM.docx]

**Supplementary Table 1: Characteristics of PWUD who were screened and not screened during their visit to the Waingmaw AHRN clinic during the survey period**

| **Characteristics at registration** | **Total** | **col %** | **Not screened** | **col %** | **Screened** | **col%** | **p value** |
| --- | --- | --- | --- | --- | --- | --- | --- |
| **Total** | 1,237 |  | 820 |  | 417 |  |  |
| Age, mean (SD) | 33 (9.2) |  | 34 (9.5) |  | 32 (8.4) |  | **0.002** |
| **Education** |  |  |  |  |  |  |  |
| Illiterate | 77 | 6.2% | 57 | 7.0% | 20 | 4.8% | **0.006** |
| Read/Write | 13 | 1.1% | 10 | 1.2% | 3 | 0.7% |  |
| Primary school | 278 | 22.5% | 205 | 25.0% | 73 | 17.5% |  |
| Middle school | 502 | 40.6% | 317 | 38.7% | 185 | 44.4% |  |
| High school | 284 | 23.0% | 177 | 21.6% | 107 | 25.7% |  |
| University Level | 60 | 4.9% | 34 | 4.1% | 26 | 6.2% |  |
| Not Recorded | 23 | 1.9% | 20 | 2.4% | 3 | 0.7% |  |
| **Marital status** |  |  |  |  |  |  |  |
| Single | 347 | 28.1% | 213 | 26.0% | 134 | 32.1% | 0.071 |
| Married | 331 | 26.8% | 223 | 27.2% | 108 | 25.9% |  |
| Divorced | 61 | 4.9% | 36 | 4.4% | 25 | 6.0% |  |
| Widowed | 16 | 1.3% | 10 | 1.2% | 6 | 1.4% |  |
| Not recorded | 482 | 39.0% | 338 | 41.2% | 144 | 34.5% |  |
| **Heroin use** |  |  |  |  |  |  |  |
| Yes | 1,202 | 97.2% | 793 | 96.7% | 409 | 98.1% | 0.098 |
| No | 34 | 2.7% | 27 | 3.3% | 7 | 1.7% |  |
| Not recorded | 1 | 0.1% | 0 |  | 1 | 0.2% |  |
| **ATS use** |  |  |  |  |  |  |  |
| Yes | 602 | 48.7% | 393 | 47.9% | 209 | 50.1% | 0.278 |
| No | 634 | 51.3% | 427 | 52.1% | 207 | 49.6% |  |
| Not recorded | 1 | 0.1% | 0 |  | 1 | 0.2% |  |
| **Injecting drug use** |  |  |  |  |  |  |  |
| Yes | 1,038 | 83.9% | 683 | 83.3% | 355 | 85.1% | 0.244 |
| No | 198 | 16.0% | 137 | 16.7% | 61 | 14.6% |  |
| Not recorded | 1 | 0.1% | 0 | 0.0% | 1 | 0.2% |  |

ATS = amphetamine type stimulants, SD = standard deviation, col% = column percentage,

**Supplementary table 2: Factors associated with symptoms of depression among survey clients (n=406)**

| **Characteristics** | **Total** | **Col %** | **Depression** δδ | | **Unadjusted** | | | **Migrant^** | | **Economic migration ¥** | | **Forced displacement** | |
| --- | --- | --- | --- | --- | --- | --- | --- | --- | --- | --- | --- | --- | --- |
|  |  |  | **n** | **Row %** | **OR** | **(95% CI)** | | **aOR** | **(95% CI)** | **aOR** | **(95% CI)** | **aOR** | **(95% CI)** |
| **Total** | 406 |  | 161 | (39.7) |  |  | |  |  |  |  |  |  |
| **Age group** |  |  |  |  |  |  | |  |  |  |  |  |  |
| <=24 | 90 | (22.2) | 40 | (44.4) | ref |  | | ref |  | ref |  | ref |  |
| 25-34 | 182 | (44.8) | 67 | (36.8) | 0.73 | (0.44-1.22) | | 0.71 | (0.41-1.25) | 0.73 | (0.42-1.27) | 0.73 | (0.42-1.28) |
| 35-44 | 106 | (26.1) | 44 | (41.5) | 0.89 | (0.50-1.56) | | 1.02 | (0.55-1.90) | 1.04 | (0.56-1.94) | 1.05 | (0.56-1.96) |
| >=45 | 28 | (6.9) | 10 | (35.7) | 0.69 | (0.29-1.67) | | 1.11 | (0.42-2.96) | 1.02 | (0.38-2.76) | 1.24 | (0.47-3.28) |
| **Ethnicity** |  |  |  |  |  |  | |  |  |  |  |  |  |
| Non-Kachin$ | 123 | (30.3) | 52 | (42.3) | ref |  | |  |  |  |  |  |  |
| Kachin | 283 | (69.7) | 109 | (38.5) | 0.86 | (0.56-1.32) | |  |  |  |  |  |  |
| **Education** |  |  |  |  |  |  | |  |  |  |  |  |  |
| Primary school or no school | 104 | (25.6) | 33 | (31.7) | ref |  | | ref |  | ref |  | ref |  |
| Middle school | 167 | (41.1) | 64 | (38.3) | 1.34 | (0.80-2.24) | | 1.30 | (0.73-2.31) | 1.27 | (0.72-2.26) | 1.4 | (0.78-2.51) |
| High school & College | 135 | (33.3) | 64 | (47.4) | **1.94** | **(1.14-3.31)** | | 1.83 | (1.01-3.32) | 1.85 | (1.02-3.36) | 2.03 | (1.11-3.69) |
| **Main income from farming** |  |  |  |  |  |  | |  |  |  |  |  |  |
| Yes | 196 | (48.3) | 68 | (34.7) | **0.67** | **(0.45-1.00)** | | 0.68 | (0.44-1.06) | 0.66 | (0.42-1.02) | **0.64** | **(0.41-0.98)** |
| No | 210 | (51.7) | 93 | (44.3) | ref |  | | ref |  | ref |  | ref |  |
| **Type of drug use in the last 3 months** | |  |  |  |  |  | |  |  |  |  |  |  |
| ATS with or without Opioid substance | 61 | (15) | 21 | (34.4) | ref |  | |  |  |  |  |  |  |
| Opioid substances without ATS | 345 | (85) | 140 | (40.6) | 1.30 | (0.74-2.3) | |  |  |  |  |  |  |
| **Ever Injected drugs** |  |  |  |  |  |  | |  |  |  |  |  |  |
| Yes | 331 | (81.5) | 133 | (40.2) | 1.13 | (0.67-1.89) | |  |  |  |  |  |  |
| No | 75 | (18.5) | 28 | (37.3) | ref |  | |  |  |  |  |  |  |
| **Detained in the last 12months (n = 406)** | |  |  |  |  |  | |  |  |  |  |  |  |
| Yes | 30 | (7.4) | 10 | (33.3) | 0.75 | (0.34-1.64) | |  |  |  |  |  |  |
| No | 376 | (92.6) | 151 | (40.2) | ref |  | |  |  |  |  |  |  |
| **Stopped by police/ anti-drugs squad in the last 12months (n = 406)** | | | | |  | |  |  |  |  |  |  |  |
| Yes | 109 | (26.8) | 44 | (40.4) | 1.04 | (0.67-1.63) | |  |  |  |  |  |  |
| No | 297 | (73.2) | 117 | (39.4) | ref |  | |  |  |  |  |  |  |
| **Housing status (n=399)** |  |  |  |  |  |  | |  |  |  |  |  |  |
| Stable housing | 279 | (69.9) | 109 | (39.1) | ref |  | |  |  |  |  |  |  |
| Non-stable housing& | 120 | (30.1) | 49 | (40.8) | 1.08 | (0.70-1.67) | |  |  |  |  |  |  |
| **Felt hungry the last 12 months (n = 405)** | |  |  |  |  |  | |  |  |  |  |  |  |
| Yes | 112 | (27.7) | 65 | (58) | **2.84** | **(1.81-4.44)** | | **2.45** | **(1.51-3.97)** | **2.44** | **(1.49-3.99)** | **2.43** | **(1.50-3.92)** |
| No | 293 | 72.3) | 96 | (32.8) | ref |  | | ref |  | ref |  | ref |  |
| **Recent experience of emotional violence** µ **(n = 403)** | | | |  |  |  | |  |  |  |  |  |  |
| Yes | 246 | (60.7) | 114 | (46.3) | **2.02** | **(1.32-3.09)** | | 1.54 | (0.97-2.45) | 0.64 | (0.39-1.06) | 0.69 | (0.42-1.14) |
| No | 157 | 38.8) | 47 | (29.9) | ref |  | | ref |  | ref |  | ref |  |
| **Recent experience of physical violence** ¶ **(n = 404)** | | | |  |  |  | |  |  |  |  |  |  |
| Yes | 37 | (9.2) | 17 | (45.9) | **1.33** | **(0.67-2.63)** | |  |  |  |  |  |  |
| No | 367 | (90.8) | 143 | (39) | ref |  | |  |  |  |  |  |  |
| **HIV result (n = 396)** |  |  |  |  |  |  | |  |  |  |  |  |  |
| Negative | 141 | (35.6) | 62 | (44) | ref |  | |  |  |  |  |  |  |
| Positive | 255 | (64.4) | 93 | (36.5) | 0.73 | (0.48-1.11) | |  |  |  |  |  |  |
| **Had ORW visits in the last 12 months (n =392)** | | |  |  |  |  | |  |  |  |  |  |  |
| Yes | 82 | (20.9) | 29 | (35.4) | ref |  | |  |  |  |  |  |  |
| No | 310 | (79.1) | 122 | (39.4) | 1.19 | (0.71-1.97) | |  |  |  |  |  |  |
| **Type of current treatment (n = 403)** | |  |  |  |  |  | |  |  |  |  |  |  |
| No current treatment | 325 | (80.6) | 131 | (40.3) | ref |  | |  |  |  |  |  |  |
| Methadone | 40 | (9.9) | 13 | (32.5) | 0.71 | (0.35-1.43) | |  |  |  |  |  |  |
| Non-methadone treatment* | 38 | (9.4) | 16 | (42.1) | 1.08 | (0.55-2.13) | |  |  |  |  |  |  |
| **Average stigma score within family** σ **(n = 406)** | | |  |  |  |  | |  |  |  |  |  |  |
| Between 1 and 2 | 213 | (52.5) | 63 | (29.6) | ref |  | | ref |  | ref |  | ref |  |
| Between 2 and 5 | 193 | (47.5) | 98 | (50.8) | **2.46** | **(1.63-3.69)** | | **1.82** | **(1.16-2.87)** | **1.77** | **(1.12-2.79)** | **1.84** | **(1.17-2.90)** |
| **Average stigma score within health care providers** σ **(n = 406)** | | | |  |  |  | |  |  |  |  |  |  |
| Score 1 | 380 | (93.6) | 144 | (37.9) | ref |  | | ref |  | ref |  | ref |  |
| More than 1 | 26 | (6.4) | 17 | (65.4) | **3.10** | **(1.34-7.13)** | | 2.23 | (0.90-5.49) | 2.28 | (0.92-5.67) | **2.48** | **(1.00-6.13)** |
| **Being Migrant^ (n = 406)** |  |  |  |  |  |  | |  |  |  |  |  |  |
| Yes | 115 | (28.3) | 52 | (45.2) | 1.38 | (0.89-2.13) | | 1.14 | (0.70-1.85) |  |  |  |  |
| No | 291 | (71.7) | 109 | (37.5) | ref |  | | ref |  |  |  |  |  |
| **Economic migration** ¥ **(n = 398)** | |  |  |  |  |  | |  |  |  |  |  |  |
| Yes | 310 | (77.9) | 129 | (41.6) | 1.53 | (0.92-2.52) | |  |  | 1.12 | (0.64-1.95) |  |  |
| No | 88 | (22.1) | 28 | (31.8) | ref |  | |  |  | ref |  |  |  |
| **Forced displacement** £ **(n = 406)** | | |  |  |  |  | |  |  |  |  |  |  |
| Yes | 79 | (19.5) | 42 | (53.2) | **1.98** | **(1.21-3.26)** | |  |  |  |  | **2.00** | **(1.18-3.40)** |
| No | 327 | (80.5) | 119 | (36.4) | ref |  | |  |  |  |  | ref |  |

n= number, ref = reference category, col% = column percentage, Row% = row percentage, OR = unadjusted odds ratio, aOR = adjusted odds ratio, 95% CI = 95% confidence interval, ATS = amphetamine-type stimulants, HIV = Human immunodeficiency virus, HCV = Hepatitis C virus,

δδ Presence of symptoms of depression was defined having a total score ≥3 for last 2 questions of PHQ4.

$ Non-Kachin ethnicity contained Shan, Bamar, Chinese, Gawrakha, Karen, Mon, Naga, and Rakhine ethnicities.

&Non-stable housing refers to living in work-provided accommodation or living in someone's house or an internally displaced persons camp, no fixed address, drug treatment institution, drug rehabilitation centre or in jail in the last 3 months.

* Non-methadone treatment refers to religious-based psychosocial support, counselling, and treatment at NGO clinics. Methadone was provided only in the public facilities at the survey site.

σ Sigma score can be interpreted as 1 = Never, 2 = Not often, 3 = somewhat often, 4 = Often, 5 = Very Often.

^ Migrant was defined as those who had not been living in Waingmaw for their whole life, and the adjusted model used migrant as a key exposure variable.

¥ Economic migration refers to those who had ever migrated for work, and the adjusted model used economic migration as a key exposure variable.

£ Forced displacement refers to those who had ever migrated because of war or armed conflict, and the adjusted model used forced displacement as key exposure variable.

µ Recent experience of emotional violence refers to being called in a derogatory term in the last 12 months.

¶ Recent experience of physical violence refers to being physically abused in the last 12 months

**Supplementary table 3: Factors associated with symptoms of anxiety among survey clients (n=406)**

| **Characteristics** | **Total** | **Col %** | **Anxiety** δδ | | **Unadjusted** | | **Migrant^** | | **Economic migration** | | **Forced displacement** | |
| --- | --- | --- | --- | --- | --- | --- | --- | --- | --- | --- | --- | --- |
|  |  |  | **n** | **Row %** | **OR** | **(95% CI)** | **aOR** | **(95% CI)** | **aOR** | **(95% CI)** | **OR** | **(95% CI)** |
| **Total** | 406 |  | 114 | (28.1) |  |  |  |  |  |  |  |  |
| **Age group** |  |  |  |  |  |  |  |  |  |  |  |  |
| <=24 | 90 | (22.2) | 28 | (31.1) | ref |  | ref |  | ref |  | ref |  |
| 25-34 | 182 | (44.8) | 53 | (29.1) | 0.91 | (0.53-1.58) | 1.03 | (0.57-1.89) | 1.10 | (0.60-2.02) | 1.04 | (0.57-1.91) |
| 35-44 | 106 | (26.1) | 26 | (24.5) | 0.72 | (0.38-1.35) | 0.83 | (0.42-1.65) | 0.89 | (0.45-1.78) | 0.82 | (0.41-1.62) |
| >=45 | 28 | (6.9) | 7 | (25.0) | 0.74 | (0.28-1.94) | 0.98 | (0.34-2.78) | 1.07 | (0.37-3.06) | 1.00 | (0.35-2.83) |
| **Ethnicity** |  |  |  |  |  |  |  |  |  |  |  |  |
| Non-Kachin$ | 123 | (30.3) | 32 | (26.0) | ref |  |  |  |  |  |  |  |
| Kachin | 283 | (69.7) | 82 | (29.0) | 1.16 | (0.72-1.87) |  |  |  |  |  |  |
| **Education** |  |  |  |  |  |  |  |  |  |  |  |  |
| Primary school or no school | 104 | (25.6) | 28 | (26.9) | ref |  |  |  |  |  |  |  |
| Middle school | 167 | (41.1) | 47 | (28.1) | 1.06 | (0.61-1.84) |  |  |  |  |  |  |
| High school & College | 135 | (33.3) | 39 | (28.9) | 1.10 | (0.62-1.95) |  |  |  |  |  |  |
| **Main income from farming** |  |  |  |  |  |  |  |  |  |  |  |  |
| Yes | 196 | (48.3) | 67 | (34.2) | 0.67 | (0.43-1.04) |  |  |  |  |  |  |
| No | 210 | (51.7) | 47 | (22.4) | ref |  |  |  |  |  |  |  |
| **Type of drug use in the last 3 months** | |  |  |  |  |  |  |  |  |  |  |  |
| ATS with or without Opioid substance | 61 | (15) | 13 | (21.3) | ref |  |  |  |  |  |  |  |
| Opioid substances without ATS | 345 | (85) | 101 | (29.3) | 1.53 | (0.79-2.94) |  |  |  |  |  |  |
| **Ever Injected drugs** |  |  |  |  |  |  |  |  |  |  |  |  |
| Yes | 331 | (81.5) | 93 | (28.1) | 1.00 | (0.58-1.76) |  |  |  |  |  |  |
| No | 75 | (18.5) | 21 | (28.0) | ref |  |  |  |  |  |  |  |
| **Detained in the last 12months (n = 406)** | |  |  |  |  |  |  |  |  |  |  |  |
| Yes | 30 | (7.4) | 11 | (36.7) | 1.53 | (0.71-3.34) |  |  |  |  |  |  |
| No | 376 | (92.6) | 103 | (27.4) | ref |  |  |  |  |  |  |  |
| **Stopped by police/ anti-drugs squad in the last 12months (n = 406)** | | | | |  |  |  |  |  |  |  |  |
| Yes | 109 | (26.8) | 30 | (27.5) | 0.96 | (0.59-1.57) |  |  |  |  |  |  |
| No | 297 | (73.2) | 84 | (28.3) | ref |  |  |  |  |  |  |  |
| **Housing status (n=399)** |  |  |  |  |  |  |  |  |  |  |  |  |
| Stable housing | 279 | (69.9) | 76 | (27.2) | ref |  |  |  |  |  |  |  |
| Non-stable housing& | 120 | (30.1) | 34 | (28.3) | 1.06 | (0.66-1.7) |  |  |  |  |  |  |
| **Felt hungry the last 12 months (n = 405)** | |  |  |  |  |  |  |  |  |  |  |  |
| Yes | 112 | (27.7) | 56 | (50) | **4.05** | **(2.54-6.47)** | **3.50** | **(2.13-5.75)** | **3.31** | **(1.99-5.51)** | **3.55** | **(2.16-5.85)** |
| No | 293 | (72.3) | 58 | (19.8) | ref |  | ref |  | ref |  | ref |  |
| **Recent experience of emotional violence** µ **(n = 403)** | | | |  |  |  |  |  |  |  |  |  |
| Yes | 246 | (60.7) | 81 | (32.9) | **1.92** | **(1.2-3.07)** | 1.68 | (1.00-2.84) | **1.73** | **(1.02-2.94)** | 1.59 | (0.94-2.70) |
| No | 157 | (38.8) | 32 | (20.4) | ref |  | ref |  | ref |  | ref |  |
| **Recent experience of physical violence** ¶ **(n = 404)** | | | |  |  |  |  |  |  |  |  |  |
| Yes | 37 | (9.2) | 19 | (51.4) | **3.02** | **(1.52-6)** | 1.66 | (0.77-3.59) | 1.8 | (0.82-3.95) | 1.67 | (0.77-3.61) |
| No | 367 | (90.8) | 95 | (25.9) | ref |  | ref |  | ref |  | ref |  |
| **HIV result (n = 396)** |  |  |  |  |  |  |  |  |  |  |  |  |
| Negative | 141 | (35.6) | 44 | (31.2) | ref |  |  |  |  |  |  |  |
| Positive | 255 | (64.4) | 68 | (26.7) | 0.80 | (0.51-1.26) |  |  |  |  |  |  |
| **Had ORW visits in the last 12 months (n =392)** | | |  |  |  |  |  |  |  |  |  |  |
| Yes | 82 | (20.9) | 23 | (28) | ref |  |  |  |  |  |  |  |
| No | 310 | (79.1) | 85 | (27.4) | 0.97 | (0.56-1.67) |  |  |  |  |  |  |
| **Type of current treatment (n = 403)** | |  |  |  |  |  |  |  |  |  |  |  |
| No current treatment | 325 | (80.6) | 96 | (29.5) | ref |  |  |  |  |  |  |  |
| Methadone | 40 | (9.9) | 8 | (20) | 0.60 | (0.27-1.34) |  |  |  |  |  |  |
| Non-methadone treatment* | 38 | (9.4) | 9 | (23.7) | 0.74 | (0.34-1.62) |  |  |  |  |  |  |
| **Average stigma score within family** σ **(n = 406)** | | |  |  |  |  |  |  |  |  |  |  |
| Between 1 and 2 | 213 | (52.5) | 48 | (22.5) | ref |  | ref |  | ref |  | ref |  |
| Between 2 and 5 | 193 | (47.5) | 66 | (34.2) | **1.79** | **(1.15-2.77)** | 1.12 | (0.68-1.85) | 1.06 | (0.64-1.76) | 1.13 | (0.68-1.86) |
| **Average stigma score within health care providers** σ **(n = 406)** | | | |  |  |  |  |  |  |  |  |  |
| Score 1 | 380 | (93.6) | 102 | (26.8) | ref |  | ref |  | ref |  | ref |  |
| More than 1 | 26 | (6.4) | 12 | (46.2) | **2.34** | **(1.05-5.22)** | 1.58 | (0.65-3.82) | 1.56 | (0.63-3.88) | 1.65 | (0.68-4.00) |
| **Being Migrant^ (n = 406)** |  |  |  |  |  |  |  |  |  |  |  |  |
| Yes | 115 | (28.3) | 38 | (33) | 1.4 | (0.87-2.23) | 1.2 | (0.72-1.99) |  |  |  |  |
| No | 291 | (71.7) | 76 | (26.1) | ref |  | ref |  |  |  |  |  |
| **Economic migration** ¥ **(n = 398)** | |  |  |  |  |  |  |  |  |  |  |  |
| Yes | 310 | (77.9) | 91 | (29.4) | 1.51 | (0.86-2.65) |  |  | 1.17 | (0.64-2.15) |  |  |
| No | 88 | (22.1) | 19 | (21.6) | ref |  |  |  | ref |  |  |  |
| **Forced displacement** £ **(n = 406)** | | |  |  |  |  |  |  |  |  |  |  |
| Yes | 79 | (19.5) | 31 | (39.2) | **1.90** | **(1.13-3.18)** |  |  |  |  | 1.69 | (0.97-2.94) |
| No | 327 | (80.5) | 83 | (25.4) | ref |  |  |  |  |  | ref |  |

n= number, ref = reference category, col% = column percentage, Row% = row percentage, OR = unadjusted odds ratio, aOR = adjusted odds ratio, 95% CI = 95% confidence interval, ATS = amphetamine-type stimulants, HIV = Human immunodeficiency virus, HCV = Hepatitis C virus,

δδ Presence of symptoms of anxiety was defined as having a total score ≥3 for first 2 questions of PHQ4.

$ Non-Kachin ethnicity contained Shan, Bamar, Chinese, Gawrakha, Karen, Mon, Naga, and Rakhine ethnicities.

&Non-stable housing refers to living in work-provided accommodation or living in someone's house or an internally displaced persons camp, no fixed address, drug treatment institution, drug rehabilitation centre or in jail in the last 3 months.

* Non-methadone treatment refers to religious-based psychosocial support, counselling, and treatment at NGO clinics. Methadone was provided only in the public facilities at the survey site.

σ Sigma score can be interpreted as 1 = Never, 2 = Not often, 3 = somewhat often, 4 = Often, 5 = Very Often.

^ Migrant was defined as those who had not been living in Waingmaw for their whole life, and the adjusted model used migrant as a key exposure variable.

¥ Economic migration refers to those who had ever migrated for work, and the adjusted model used economic migration as a key exposure variable.

£ Forced displacement refers to those who had ever migrated because of war or armed conflict, and the adjusted model used forced displacement as key exposure variable.

µ Recent experience of emotional violence refers to being called in a derogatory term in the last 12 months.

¶ Recent experience of physical violence refers to being physically abused in the last 12 months
